# Identifying the Internalizing Disorder Clusters Among Recently Hospitalized Cardiovascular Disease Patients: A Receiver Operating Characteristics Study

**DOI:** 10.3389/fpsyg.2019.02829

**Published:** 2019-12-17

**Authors:** Megan Grech, Deborah A. Turnbull, Gary A. Wittert, Phillip J. Tully, the CHAMPS Investigators, John D. Horowitz

**Affiliations:** School of Medicine, The University of Adelaide, Adelaide, SA, Australia; Department of Cardiology, Basil Hetzel Institute, Queen Elizabeth Hospital, Woodville, SA, Australia; Discipline of Psychiatry, Basil Hetzel Institute, Queen Elizabeth Hospital, The University of Adelaide, Adelaide, SA, Australia; University Hospital of Psychiatry and Psychotherapy, University of Münster, Münster, Germany; Department of Clinical Psychology and Psychotherapy, Institute of Psychology and Education, Ulm University, Ulm Germany; Center for Anxiety & Related Disorders, Department of Psychology, Boston University, Boston, MA, United States; ^1^School of Psychology, The University of Adelaide, Adelaide, SA, Australia; ^2^Freemasons Foundation Centre for Men’s Health, School of Medicine, The University of Adelaide, Adelaide, SA, Australia

**Keywords:** depression, anxiety, internalizing disorders, receiver operating characteristics, cardiovascular disease, post-traumatic stress disorder

## Abstract

Depression and anxiety disorders are common among cardiovascular disease (CVD) populations, leading several cardiology societies to recommend routine screening to streamline psychological interventions. However, it remains poorly understood whether routine screening in CVD populations identifies the broader groups of disorders that cluster together within individuals, known as anxious-misery and fear. This study examines the screening utility of four anxiety and depression questionnaires to identify the two internalizing disorder clusters; anxious-misery and fear. Patients with a recent hospital admission for CVD (*n* = 85, 69.4% males) underwent a structured clinical interview with the MINI International Neuropsychiatric Interview. The participants also completed the Patient Health Questionnaire (PHQ-9), Generalized Anxiety Disorder (GAD-7) scale, Overall Anxiety Severity Impairment Scale (OASIS), and the stress subscale of the Depression Anxiety Stress Scale (DASS). The PHQ-9 and the GAD-7 yielded appropriate screening properties to detect three different iterations of the anxious-misery cluster (sensitivity >80.95% and specificity >82.81%). The GAD-7 was the only instrument to display favorable screening properties to detect a fear cluster omitting post-traumatic stress disorder (PTSD) but including obsessive-compulsive disorder (OCD; sensitivity 81.25%, specificity 76.81%). These findings indicate that the PHQ-9 and GAD-7 could be implemented to reliably screen for anxious-misery disorders among CVD in-patients, however, the receiver operating characteristics (ROC) to detect fear disorders were contingent on the placement of PTSD and OCD within clusters. The findings are discussed in relation to routine screening guidelines in CVD populations and contemporary understandings of the internalizing disorders.

## Introduction

Depression and anxiety disorders are prevalent in between 15 and 20% of cardiovascular disease (CVD) patients, representing a substantial morbidity burden globally (Rutledge et al., 2006; Thombs et al., 2008; Magyar-Russell et al., 2011; Tully et al., 2014). The presence of depression and anxiety disorders results in substantial individual, societal and economic cost worldwide, disability, and a reduction in quality of life (Scott et al., 2009; Dickens et al., 2012; Vos et al., 2012; Baumeister et al., 2015; Chisholm et al., 2016). Moreover, depression and anxiety portend a poorer cardiovascular prognosis as independent (Rutledge et al., 2006; Thombs et al., 2008; Roest et al., 2010; Tully et al., 2014) and additive or comorbid psychiatric risk factors (Phillips et al., 2009; Watkins et al., 2013). The high prevalence of depression and anxiety disorders, coupled with adverse cardiovascular prognosis, led several cardiology societies to recommend routine screening in order to better identify and streamline psychological treatments in CVD populations in the United States (Lichtman et al., 2008), Germany (Ladwig et al., 2014), Italy (Sommaruga et al., 2018), Australia (Colquhoun et al., 2013), and Europe (Albus et al., 2004).

The identification of psychiatric disorders in CVD populations is typically based on screening for depression (Thombs et al., 2013), with less attention paid to anxiety disorders and the comorbidity between depression and anxiety disorders. One limitation of this approach is that depression and anxiety disorders are comorbid in up to 50% of patients (Kessler et al., 2005, 2012; Slade and Watson, 2006; Beesdo-Baum et al., 2009) including among CVD populations (Serber et al., 2009; Tully et al., 2014). In fact, a robust body of empirical research indicates that common mental disorders tend to cluster together, evidenced by the higher than chance comorbidity patterns observed across the lifespan (Kessler et al., 2011). In particular, the depression and anxiety disorders cluster together under a higher-order internalizing domain, which is distinct from the externalizing domain reflecting the antisocial and substance use disorders (Krueger, 1999). Subsequent research demonstrates that the internalizing domain can bifurcate into two lower order groups characterized by anxious-misery [e.g., Major Depressive Disorder, Dysthymia, Generalized Anxiety Disorder (GAD), and Post Traumatic Stress Disorder (PTSD)], or, by fear [e.g., Panic Disorder, Agoraphobia, Specific Phobia, Social Anxiety Disorder, and Obsessive-Compulsive Disorder (OCD)] (Slade et al., 2009; Watson, 2009; Eaton et al., 2013; Waszczuk et al., 2017). Notably, GAD is considered to be a part of the anxious-misery cluster compared to the other anxiety disorders subsumed under fear, which are generally characterized by phobias and somatic arousal (Watson, 2005).

Evidence indicates that the arrangement of disorders into anxious-misery and fear, in contrast to disorder-specific models, informs the prediction of CVD and other health outcomes (Eaton et al., 2013; Tully et al., 2015b; Roest et al., 2017). However, this framework is lesser utilized to inform screening procedures in health settings such as cardiology where routine depression screening is recommended. Given that comorbidity between disorders is the norm rather than the exception, it may be inappropriate to limit assessment and mental health triage to single disorders (Tully et al., 2015a). Indeed, disorder-specific screening in CVD populations omits a substantial number of persons that are candidates for intervention (Bunevicius et al., 2013). It is therefore likely that enquiries about single disorders are less meaningful when the primary goal is to detect clinically relevant psychological distress and streamline patients into clinical supports or treatments. Due to the high likelihood of comorbidity and shift toward transdiagnostic treatment approaches (Barlow et al., 2017), grouping disorders into clusters may aid screening efforts in CVD populations by cutting across discrete single-disorder categories. The current study aims to evaluate the receiver operating characteristics (ROC) of four common clinical tools for the screening of the emotional disorders in a CVD population.

We theorized that the measures designed to capture depression and GAD would have higher ROCs to detect the anxious-misery cluster than measures designed to capture phobia and avoidance. Conversely, we theorized that the measures designed to capture phobia and avoidance would have higher ROCs to detect the fear cluster than measures designed to capture depression and GAD. Measures with a high negative affectivity component were theorized to equally predict anxious-misery and fear clusters.

## Materials and Methods

### Participants

**FIGURE 1:**
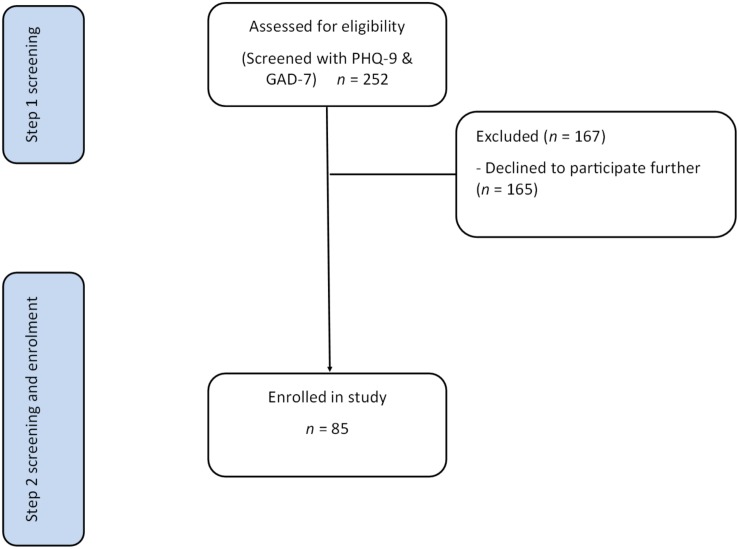
A flow chart of participant eligibility through the study. Step 1 screening was performed in hospital. Step 2 screening was performed 2 weeks after the index CVD admission. CVD, cardiovascular disease; GAD-7, Generalized Anxiety Disorder scaIe-7; PHQ-9, Patient Health Questionnaire scale-9.

Data for this study were obtained from a single-blind randomized control trial to evaluate the feasibility of the unified protocol for the transdiagnostic treatment of emotional disorders in patients recently hospitalized for CVD (Tully et al., 2016). Participant recruitment took place from January 2016 until March 2017 at the Queen Elizabeth Hospital, a tertiary hospital in the Western urban area of Adelaide, South Australia. Eligibility criteria were: a primary hospital admission for CVD specified by relevant International Classification of Disease codes (myocardial infarction, heart failure, atrial fibrillation or other ventricular or atrial arrhythmia, coronary revascularization intervention; symptomatic coronary heart disease including unstable angina pectoris, or heart valve disease); spent two or more nights admitted to the Department of Cardiology; ≥18 years of age, and proficiency in the English language. Ineligible participants had: a known or observed cognitive impairment or dementia; a medical condition likely to be fatal within 1 year; or a neurodegenerative condition such as Parkinson’s or Multiple Sclerosis (Flow chart in Figure 1). Patients with psychosis, bi-polar disorder, substance or alcohol dependence/abuse, or high suicide risk were excluded from the main trial but included in the current analyses. The rationale is that undocumented severe psychiatric disorders would be a related or incidental finding from depression and anxiety screening. The Human Research Ethics Committee from the Queen Elizabeth Hospital gave approval for the study (approval #HREC/15/TQEH47).

### Measures

#### Psychiatric Diagnosis

All participants underwent a structured diagnostic interview with a graduate from a 4th year psychology degree to determine disorder status. The assessor was trained, and supervised, in all aspects of clinical assessments. The MINI International Neuropsychiatric Interview (MINI) version 5.0.0 served as the gold standard. Analysis of previous MINI versions suggested high sensitivity and specificity to detect the emotional disorders specified by Diagnostic and Statistical Manual of Mental Disorders (DSM), and good inter-rater agreement (κ = 0.86–0.96) (Lecrubier et al., 1997; Sheehan et al., 1997, 1998; Amorim et al., 1998). The MINI modules utilized in this study covered: Major Depressive Disorder; Major Depressive Disorder with Melancholic Features, Dysthymia, Hypomanic and Manic Episode (Bipolar I and II), Panic Disorder, Agoraphobia, Social Anxiety Disorder, OCD, PTSD, Alcohol Dependence/Abuse, Substance Dependence/Abuse (non-alcohol), Psychotic Disorders, Mood Disorders with Psychotic Features, GAD, and Antisocial Personality Disorder. Specific Phobias are not assessed by the MINI 5.0.0. The modules relating to anorexia and bulimia nervosa were omitted for brevity.

#### Disorder Arrangement Into Clusters

Because of uncertainty in the optimal placement of certain disorders within the anxious-misery and fear clusters, several models were arranged and tested. Specifically, discrepant findings have been reported for the placement of OCD, PTSD, and social anxiety disorder in cross-sectional and longitudinal evaluations (Watson, 2009; Naragon-Gainey et al., 2016; Forbes et al., 2017; Kotov et al., 2017; Waszczuk et al., 2017; Conway and Brown, 2018).

Model^a^: Anxious-misery^a^ – major depressive disorder, dysthymia, GAD, PTSD, and bi-polar disorder; fear^a^ – panic disorder, agoraphobia, social anxiety disorder, and OCD.

Model^b^: This model was a variant of Model^a^ whereby PTSD was included in each cluster, with OCD included only in anxious-misery. Anxious-misery^b^ – major depressive disorder, dysthymia, GAD, major depressive disorder with melancholic features, PTSD, bi-polar disorder, and OCD; fear^b^ – panic disorder, agoraphobia, social anxiety disorder, and PTSD.

Model^c^: This model was a variant of Model^a^ and Model^b^ whereby PTSD and OCD were excluded from all clusters, in accordance with DSM-5 arrangement of PTSD and OCD as separate disorders from anxiety. Anxious-misery^c^ – major depressive disorder, dysthymia, GAD, and major depressive disorder with melancholic features; fear^c^ – panic disorder, agoraphobia, and social anxiety disorder.

#### Self-Reported Distress Scales

Participants completed a battery of self-report measures consisting of four depression and anxiety questionnaires. The GAD-7 approximates DSM-5 criteria for GAD (Spitzer et al., 2006). The GAD-7 is scored on a scale of 0 to 3 (not at all, several days, more days than half the days, and nearly every day). The GAD-7 is considered a psychometrically sound measure to use in primary care settings for detection of anxiety disorders as the measure does not contain any items tapping into somatic symptoms (Spitzer et al., 2006).

The PHQ-9 is a standardized instrument that approximates DSM-5 major depression criteria (Kroenke et al., 2001). Each item of the PHQ-9 is scored from 0 to 3, with scores ranging from 0 to 27. Scores of 10, 15, and 20 represent the thresholds for moderate-, moderately severe-, and severe-depression, respectively (Kroenke et al., 2001). The PHQ-9 is recommended for depression screening in CVD populations because of its specificity to detect depression and sensitivity to clinical change (Lichtman et al., 2008; Colquhoun et al., 2013; Ladwig et al., 2014).

The Overall Anxiety Severity and Impairment Scale (OASIS) was developed as a self-report measure that assesses clinical severity and functional impairment of anxiety disorders (Norman et al., 2006; Campbell-Sills et al., 2009). Participants respond to the items that best describe their experience on a five-point scale (0, little or none; 1, mild; 2, moderate; 3, severe; 4, extreme). The OASIS psychometric properties were evaluated in primary care settings and psychiatric samples and the measure is commonly utilized for clinical trials (Campbell-Sills et al., 2009).

Stress was measured using the stress subscale of the Depression, Anxiety and Stress Scales (DASS-21). The DASS-21 is a commonly utilized measure and validated in adults aged to 90 years (Lovibond, 1998). Scores range from 0 to 21 for the stress subscale. The DASS-21 factor structure approximates a tripartite structure (Clark and Watson, 1991), with stress broadly indicative of negative affectivity (Brown et al., 1997).

### Procedure

The assessment of depression and anxiety was based on a two-stage screening process as recommended by the American Heart Association (Lichtman et al., 2008) to confirm elevated symptoms of anxiety and depression after hospitalization. During the CVD hospital admission, each participant completed the PHQ-9 and the GAD-7 in the cardiology department. All patients were followed-up for a repeat screening approximately 2 weeks later with the PHQ-9, GAD-7, OASIS, DASS-stress, and the MINI. Patients with a PHQ-9 total score ≥10 and/or GAD-7 total score ≥7 on both occasions, and a MINI depression or anxiety disorder, were deemed eligible for the feasibility study (Tully et al., 2016). Otherwise, participants were included in a non-distressed control group. The current study utilizes only the measurements obtained 2 weeks after the CVD admission, which took place in the outpatient setting.

### Statistical Analysis

Statistical analyses were performed using MedCalc Statistical Software version, 18.5 (MedCalc Software, Ostend, Belgium). The MINI diagnosis (yes/no) constituted the criterion standard for the presence or absence of disorders. Scores on the screening measures (PHQ-9, GAD-7, OASIS, and DASS-stress), were used to detect clusters arranged into anxious-misery and fear in three iterations (denoted^a,b,c^). The ROCs were modeled to identify the true positive rate (sensitivity) plotted against the false positive rate (1-specificity) for all possible cut off points. The area under the curve (AUC) is the percentage of randomly drawn pairs for which the screening measures correctly classifies affected and non-affected cases and represents the diagnostic power of the test. An AUC of 1.0 indicates the measure has perfect diagnostic detection properties and an AUC of 0.5 indicates that the screening measure is no better than chance. Interpretation of the AUC values were as follows: 0.5 – <0.7 mildly accurate, 0.7 – 0.9 moderately accurate, and 0.9 – <1 highly accurate (Hanley and Mcneil, 1982). The screening measures cut off points were reported for AUC *p* < 0.05 and were determined by the maximal Youden Index (sensitivity + specificity – 100). The positive predictive value (PPV) is the likelihood that there is a cluster present given a positive test result, and the negative predictive value (NPV) is the likelihood that a cluster is not present given a negative test result. High sensitivity at the expense of low specificity results in an inordinate number of diagnostic interviews for false positives and therefore, a specificity of >75% is desirable for clinical purposes (Hanley and Mcneil, 1982). The AUCs between measures were compared statistically using the methods of Delong et al. (1988). Sensitivity analyses were performed excluding persons receiving psychotropic drugs or psychiatrist or psychologist care. The rationale was that depression and anxiety screening intends to identify new and previously unidentified cases.

In all analyses a *p*-value < 0.05 was considered as statistically significant, and no adjustment was made for multiple comparisons based on the recommendations of Rothman (1990). The rationale was that the study hypotheses are well defined, and secondly, that the study is exploratory in nature where the risk of Type II error is greater than the risk of Type I error. A sample size of 46 patients per group (i.e., yes/no for anxious-misery or fear) would provide 80% power to detect a θ = 0.20 difference between ROCs at α < 0.05.

## Results

A total of *n* = 85 patients were included in the current analyses. The most common CVD-cause admissions were for angina pectoris (34.1%), followed by arrhythmia (25.9%), and heart failure (22.4%) (Table 1). The number of patients diagnosed with discrete internalizing disorders on the MINI were as follows: major depressive disorder (*n* = 20, 23.5%), major depressive disorder with melancholic features (*n* = 11), GAD (*n* = 7, 8.2%), agoraphobia (*n* = 9, 10.6%), panic disorder (*n* = 6, 7.1%), bipolar depression (*n* = 4, 4.7%), social phobia (*n* = 2, 2.4%), PTSD (*n* = 2, 2.4%), OCD (*n* = 1, 1.2%). There were no dysthymia cases due to hierarchal exclusion rules (*n* = 0). In regards to comorbidity, the number of patients with comorbid affective disorders were as follows: no disorder (*n* = 57, 67.1%), one disorder (*n* = 12, 14.1%), two disorders (*n* = 4, 4.7%), three disorders (*n* = 7, 8.2%), four disorders (*n* = 3, 3.5%), six disorders (*n* = 1, 1.2%), and seven disorders (*n* = 1, 1.2%). The number of patients diagnosed with discrete externalizing disorders on the MINI were as follows: manic episode (*n* = 2, 2.4%), hypomanic episode (*n* = 3, 3.5%), alcohol dependence (*n* = 3, 3.5%), alcohol abuse (*n* = 1, 1.2%), psychotic disorder (*n* = 1, 1.2%), mood disorder with psychotic features (*n* = 1, 1.2%). No patients met criteria for substance abuse or dependence. On the MINI, suicide risk was rated as moderate for nine patients (10.6%) and high for four patients (4.7%).

**TABLE 1:** Descriptive and medical comorbidity data.

**Descriptive variables**	**N (%)**
Male sex	59 (69.4)
Age in years, M ± SD	63.3 ± 11.6
**Employment status**	
Working	44 (51.8)
Retired	22 (25.9)
Unemployed	2 (2.4)
**Primary CVD admission cause**	
Acute myocardial infarction	11 (12.9)
Heart failure	19 (22.4)
Arrhythmia	22 (25.9)
Revascularization	2 (2.4)
Angina pectoris with CAD	29 (34.1)
Other CVD	2 (2.4)
**CVD comorbidities**	
Past myocardial infarction	18 (21.2)
Past revascularization	10 (11.8)
Valve disease	8 (9.4)
Biventricular pacemaker	5 (5.9)
Implantable cardioverter defibrillator	7 (8.2)
Stroke or cerebrovascular accident	8 (9.4)
Lung disease	9 (10.6)
Renal disease	8 (9.4)
Diabetes	22 (25.9)
Hypertension	56 (65.9)
Hypercholesterolemia	48 (56.5)
Tobacco smoking (current)	5 (5.9)
Sleep apnea	14 (16.5)
Chronic pain	20 (23.5)
**Psychiatric treatment**	
Using SSRI or SNRI	2 (2.4)
Using TCA	2 (2.4)
Using anxiolytic	1 (1.2)
Current psychiatrist care	1 (1.2)
Current psychologist care	–

*CAD, coronary artery disease; CVD, cardiovascular disease; SNRI, serotonin and norepinephrine reuptake inhibitors; SSRI, selective serotonin reuptake inhibitors; TCA, tricyclic antidepressants.*

### Receiver Operating Characteristics

**FIGURE 2:**
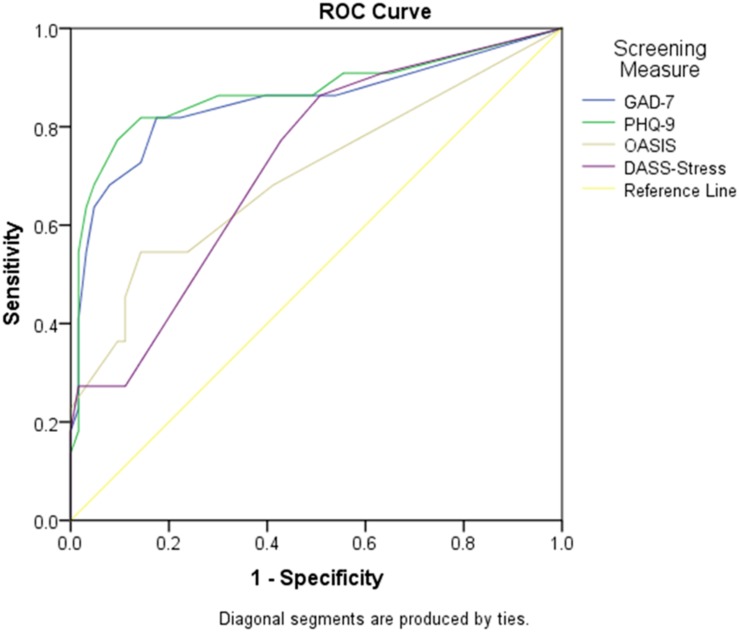
Screening measure AUC to detect anxious-misery disorders. Graph showing the AUC (sensitivity and 1- specificity) for the GAD-7, PHQ-9, OASIS, and DASS-stress scales to detect the anxious-misery*^*a*^* cluster (major depressive disorder, dysthymia, generalized anxiety disorder, post-traumatic stress disorder, bipolar disorder). AUC, area under the curve; DASS-stress, Depression, Anxiety and Stress Scales-stress subscale; GAD-7, Generalized Anxiety Disorder 7 item scale; OASIS, Overall Anxiety Severity and Impairment Scale; PHQ-9, Patient Health Questionnaire scale 9 item scale.

*Anxious-misery cluster^*a*^* (*n* = 21, 24.7%): The ROCs are presented in Table 2. The AUC was greatest for the PHQ-9, followed by the GAD-7, the DASS-stress, and the OASIS. Using a cut-point of six, the PHQ-9 showed favorable sensitivity (85.71%) and specificity (82.94%). Employing a cut point of 4, the GAD-7 yielded comparable sensitivity (85.71%) and specificity (82.81%). The DASS-stress scale and OASIS sensitivity and specificity rates were suboptimal for screening purposes to detect the anxious-misery^a^ cluster. When AUCs were compared, the PHQ-9 (*p* = 0.049) and the GAD-7 (*p* = 0.048) had significantly higher AUCs than the OASIS (Figure 2).

**TABLE 2:** Receiver operating characteristics of depression and anxiety screening measures to detect anxious-misery and fear clusters.

**Clusters**	**AUC (SE)**	**95% CI**	**Cut-off**	**Sensitivity true +**	**Specificity true −**	**Youden index**	**PPV**	**NPV**
**Anxious-misery^a^ (*n* = 21)**								
GAD-7^1^	0.856 (0.060)	0.763–0.923	4	85.71	82.81	68.53	55.5	95.9
PHQ-9^2^	0.873 (0.058)	0.783–0.935	6	85.71	85.94	71.65	66.7	94.8
OASIS	0.692 (0.72)	0.582–0.787	2	52.38	84.37	36.75	52.4	84.4
DASS	0.732 (0.60)	0.625–0.822	2	80.95	57.81	38.76	38.6	90.2
**Anxious-misery^b^ (*n* = 21)**								
GAD-7^1^	0.860 (0.060)	0.758–0.930	4	85.71	84.31	70.03	57.5	95.8
PHQ-9^2^	0.879 (0.057)	0.780–0.944	7	80.95	94.12	75.07	77.5	95.2
OASIS	0.680 (0.074)	0.560–0.785	3	47.62	88.24	35.85	50.3	87.1
DASS	0.747 (0.061)	0.631–0.842	2	80.95	62.75	43.70	35.2	92.9
**Anxious-misery^c^ (*n* = 21)**								
GAD-7^1^	0.856 (0.060)	0.763–0.923	4	85.71	82.81	68.53	55.5	95.9
PHQ-9^2^	0.873 (0.058)	0.783–0.935	6	85.71	85.94	71.65	60.4	96.0
OASIS	0.692 (0.072)	0.582–0.787	2	52.38	84.37	36.76	45.6	87.6
DASS	0.732 (0.060)	0.625–0.822	2	80.95	57.81	38.76	32.4	92.4
**Fear^a^ (*n* = 17)**								
GAD-7^3^	0.776 (0.076)	0.673–0.860	4	81.25	76.81	58.06	38.2	95.9
PHQ-9	0.719 (0.082)	0.611–0.811	7	68.75	82.61	51.36	41.1	93.7
OASIS	0.673 (0.073)	0.563–0.771	0	75.00	57.97	32.97	29.3	90.9
DASS	0.626 (0.077)	0.474–0.777	2	75.00	53.62	28.62	22.2	92.4
**Fear^b^ (*n* = 16)**								
GAD-7	0.787 (0.077)	0.684–0.868	7	68.75	91.30	60.05	58.3	94.3
PHQ-9	0.732 (0.084)	0.625–0.823	7	68.75	82.61	51.36	41.1	93.7
OASIS	0.669 (0.072)	0.559–0.768	0	75.00	57.97	32.97	23.9	92.9
DASS	0.621 (0.074)	0.510–0.724	2	75.00	53.62	28.62	22.2	92.4
**Fear^c^ (*n* = 15)**								
GAD-7^3^	0.768 (0.080)	0.663–0.852	7	66.67	90.00	56.67	54.1	93.9
PHQ-9	0.710 (0.087)	0.601–0.803	7	66.67	81.43	48.10	38.8	93.3
OASIS	0.642 (0.074)	0.531–0.743	0	73.33	57.14	30.48	23.2	92.4
DASS	0.592 (0.075)	0.480–0.698	2	73.33	52.86	26.19	21.5	91.8

*AUC, area under the curve; CI, confidence interval; NPV, negative predictive value; PPV, positive predictive value; SE, standard error; GAD-7, Generalized Anxiety Disorder-7 scale; PHQ-9, Patient Health Questionnaire-9 scale; OASIS, Overall Anxiety Severity Impairment Scale; DASS, Depression Anxiety Stress Scale – Stress subscale. Anxious-misery^*a*^ – major depression, dysthymia, generalized anxiety disorder, depression melancholic, post-traumatic stress, and bi-polar; Fear^*a*^ – panic disorder, agoraphobia, social anxiety disorder, and obsessive-compulsive disorder. Anxious-misery^*b*^ – major depression, dysthymia, generalized anxiety disorder, depression melancholic, bi-polar and obsessive-compulsive disorder; Fear^*b*^ – disorder, agoraphobia, social anxiety disorder, and post-traumatic stress disorder. Anxious-misery^*c*^ – major depression, dysthymia, generalized anxiety disorder, and depression melancholic; Fear^*c*^ – panic disorder, agoraphobia, and social anxiety disorder. 1 – The GAD-7 was significantly different (*p* < 0.05) from the OASIS; 2 – The PHQ-9 was significantly different (*p* < 0.05) from the OASIS; 3– The GAD-7 was significantly different (*p* < 0.05) from the DASS-stress.*

*Anxious-misery cluster^*b*^* (*n* = 21, 24.70%): Employing a cut off of 7 the PHQ-9 showed desirable sensitivity (80.95%) and specificity (94.12%), while the GAD-7 required a cut point of four for a sensitivity of 85.71% and specificity of 84.31%. The DASS-stress scale and the OASIS, again, demonstrated suboptimal screening properties in the detection of anxious-misery^b^. *Post hoc* tests revealed that the GAD-7 (*p* = 0.031) and PHQ-9 (*p* = 0.031) AUCs were both statistically different from the OASIS.

*Anxious-misery cluster^*c*^* (*n* = 21, 24.70%): Again, the PHQ-9 showed favorable sensitivity (85.71%) and specificity (82.94%). Employing a cut point of 4 the GAD-7 also yielded a highly favorable sensitivity of 80.95% and a specificity of 94.12%. The GAD-7 (*p* = 0.048) and PHQ-9 (*p* = 0.049) AUCs were statistically significant when compared to the AUC of the OASIS.

**FIGURE 3:**
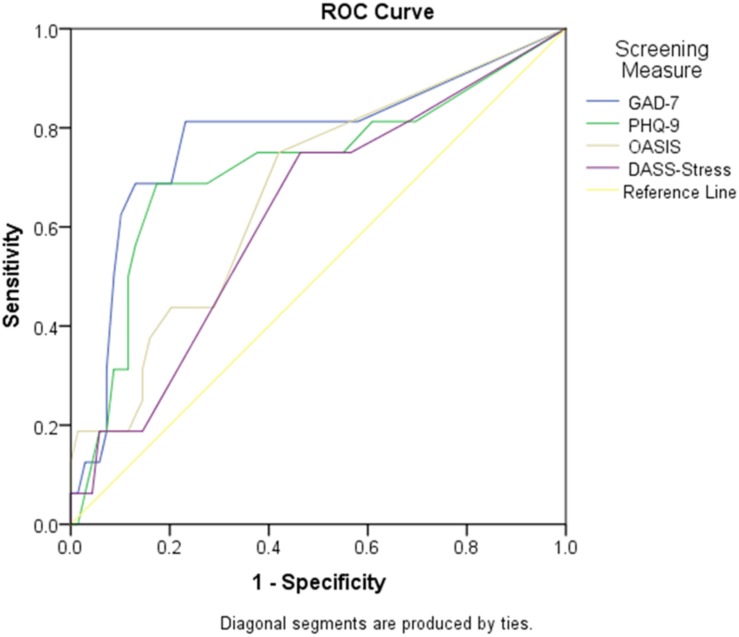
Screening measure AUC to detect fear disorders. Graph showing the AUC (sensitivity and 1- specificity) for the GAD-7, PHO-9, OASIS, and DASS-stress scales to detect the fear*^*a*^* cluster (panic disorder, agoraphobia, social anxiety disorder, obsessive-compulsive disorder). AUC, area under the curve; DASS-stress, Depression, Anxiety and Stress Scales-stress subscale; GAD-7, Generalized Anxiety Disorder 7 item scale; OASIS, Overall Anxiety Severity and Impairment Scale.

*Fear cluster^*a*^* (*n* = 17, 21.52%): The GAD-7 was the only measure to demonstrate suitable sensitivity and specificity values for the fear^a^ cluster. The AUC was greatest for the GAD-7 employing a cut of four, with favorable sensitivity (81.25%) and specificity (76.81%). A cut-point of seven on the PHQ-9 showed a sensitivity of 68.75% and a specificity of 82.61% and therefore, considered unfavorable for screening purposes. Further, the OASIS and the DASS-stress yield matching sensitivity scores (75%) but unfavorable specificity values (57.97% and 53.62%, respectively). The GAD-7 AUC was statistically significantly different from the DASS-stress scale (*p* = 0.046) (Figure 3).

*Fear cluster^*b*^* (*n* = 16, 18.82%): The GAD-7 was no longer considered to be an appropriate screener for fear cluster^b^ due to low sensitivity (68.75%), though the GAD-7 was statistically different from the DASS-stress scale (*p* = 0.027). Irrespective, no measure was considered appropriate for detection of fear cluster^b^.

*Fear cluster^*c*^* (*n* = 15, 17.6%): Both the GAD-7 and the PHQ-9 yielded unfavorable sensitivity scores (sensitivity, 66.67%), while the OASIS and the DASS-stress scale had specificity values considered to be suboptimal (specificity, <70). The GAD-7 was considered to be diagnostically more accurate than the DASS-stress scale in fear cluster^c^ disorders (*p* = 0.024). Despite this finding, no measures in this cluster had suitable screening properties when evaluating the sensitivity and specificity values.

### Sensitivity Analysis

After excluding persons receiving current psychiatric treatment, similar findings were observed for detecting the anxious-misery cluster (Table 3). The PHQ-9 and GAD-7 revealed favorable sensitivity (82.35% and 70.59%, respectively) and specificity (90.32% and 95.16%, respectively). The AUC for the PHQ-9 (*p* = 0.043) was significantly different from the DASS-stress scale indicating further screening benefits of the PHQ-9. The GAD-7 was the only screening measure from the fear^a^ cluster with favorable diagnostic qualities (sensitivity, 78.57%; specificity, 78.46%). Comparison of ROC curves did not yield any statistically significant differences between the screening measures suggesting similar screening accuracy across outcomes.

**TABLE 3:** Receiver operating characteristics from sensitivity analyses excluding persons receiving current psychiatric treatment.

**Clusters**	**AUC (SE)**	**95% CI**	**Cut-off**	**Sensitivity true +**	**Specificity true −**	**Youden index**	**PPV**	**NPV**
**Anxious-misery^a^ (*n* = 17)**								
GAD-7	0.836 (0.719)	0.736–0.910	7	70.59	95.16	65.75	78.5	92.8
PHQ-9^1^	0.856 (0.070)	0.759–0.925	7	82.35	90.32	72.68	68.0	95.3
OASIS	0.647 (0.080)	0.531–0.751	2	47.06	87.10	34.16	47.7	86.8
DASS	0.679 (0.068)	0.565–0.780	2	76.47	56.45	32.92	28.5	91.6
**Fear^a^ (*n* = 14)**								
GAD-7	0.762 (0.084)	0.653–0.851	4	78.57	78.46	57.03	39.2	95.4
PHQ-9	0.701 (0.090)	0.588–0.799	7	64.29	83.08	47.36	40.1	92.9
OASIS	0.675 (0.077)	0.561–0.776	0	71.43	61.54	32.97	24.7	92.4
DASS	0.605 (0.081)	0.489–0.713	2	71.43	53.85	25.27	21.5	91.4

*AUC, area under the curve; CI, confidence interval; NPV, negative predictive value; PPV, positive predictive value; SE, standard error; GAD-7, Generalized Anxiety Disorder-7 scale; PHQ-9, Patient Health Questionnaire-9 scale; OASIS, Overall Anxiety Severity Impairment Scale; DASS, Depression Anxiety Stress Scale – Stress subscale. 1 – The PHQ-9 was significantly different (p < 0.05) from DASS-stress.*

## Discussion

The GAD-7 and the PHQ-9 both had favorable ROCs for screening of the anxious-misery disorders, irrespective of cluster variations. The ROCs were generally suboptimal for the detection of fear disorders, with only the GAD-7 yielding sufficient sensitivity and specificity values to detect the fear^a^ cluster. However, the ability to detect fear disorders was dependent on inclusion of OCD and the exclusion of PTSD from the fear cluster. The OASIS yielded unsuitable ROCs for screening purposes, thus not supporting our hypotheses. Taken together, the results here obtained 2 weeks after a CVD admission are promising given that the aim of screening is to distinguish patients with transient or short-term distress from those with clinically relevant disorders requiring mental health treatment.

Our results offer new information beyond studies primarily focusing on depression screening in CVDs. A systematic review of depression measures indicated favorable ROCs for most depression questionnaires to detect major and minor depression in coronary heart disease populations (Thombs et al., 2008). The current work extends beyond these previous studies by arranging disorders into theorized anxious-misery and fear clusters. The overall performance of the PHQ-9 and GAD-7 were remarkably similar in detection of the anxious-misery disorders and cluster iterations^a,b,c^. Thus, the PHQ-9 and GAD-7 ROCs were largely unrelated to the placement of PTSD and OCD within or excluded from anxious-misery. One explanation for the similarities in screening properties is that the PHQ-9 and GAD-7 cover the core symptoms of major depressive disorder and GAD and may capture a negative affectivity component related more strongly to anxious-misery (Bohnke et al., 2014).

Anxiety disorder screening in CVD populations is less common and has proven challenging to identify suitable screening measures. Bunevicius et al. (2013) showed that the Hospital Anxiety and DepressionScale-Anxiety (HADS-A), the Spielberger State-Trait Anxiety Inventory, and the Spielberger State Anxiety Inventory yielded high false positive rates for anxiety disorder screening in CVD populations. Likewise, screening measures based on a tripartite structure of depression and anxiety yielded unsuitable ROCs to detect fear disorders (Tully and Penninx, 2012). Here the only diagnostically accurate screening tool for the fear cluster was the GAD-7 and this was limited to fear^a^ (i.e., panic, agoraphobia, social anxiety, and OCD). Recently, it was suggested that worry, the hallmark feature of GAD, may be best modeled at a broad structural level, rather than an indicator of fear or anxious-misery clusters (Naragon-Gainey et al., 2016). This provides a potential explanation for the ability of the GAD-7 to screen both fear and anxious-misery clusters (Naragon-Gainey et al., 2016). Intriguingly, the removal of OCD (*n* = 1) made GAD-7 screening inadequate for fear^c^ cluster. The increase in the sensitivity of fear^a^ cluster as a result of including OCD may simply be due to some overlap in the symptoms of OCD and those captured by the GAD-7 (e.g., feeling anxious or on edge, trouble relaxing, and feeling afraid something awful might happen). In addition, worries can also be present in individuals with OCD (Abramowitz and Foa, 1998), and obsessions might be understood or subjectively perceived by patients as worries. A recent review and meta-analysis indicated that further studies are required in primary care to determine if the GAD-7 is proficient in detecting fear disorders including OCD (Plummer et al., 2016).

The finding that the OASIS screening properties were unfavorable for the detection of fear disorders did not support our hypothesis. Other work suggests that the AUC is fair for the OASIS when applied to anxiety disorders (Ito et al., 2015). An explanation for the low sensitivity and AUC produced by the OASIS here is that the measure was designed to tap the behavioral (i.e., avoidance) and functional aspects (i.e., impairment in work, or, interpersonal relationships) of disorder severity. The GAD-7 and PHQ-9 are likely affected by the frequency of cognitive-affective and/or somatic aspects of anxiety or depression (Ito et al., 2015). The symptoms experienced by CVD patients might be different and more somatically driven than the avoidance or functional impairment symptoms captured by the OASIS and those considered as hallmark features of the fear cluster (Mineka and Zinbarg, 2006). Moreover, patients with CVDs tend to experience less health-related and heart-focused anxiety by comparison to chest-pain patients without known cardiac disease (Marker et al., 2008; Robertson et al., 2008).

The hypotheses that the DASS-stress measure would be associated with both the anxious-misery and fear clusters was partially supported, albeit that the ROCs were suboptimal. The AUC was moderately accurate at detecting the fear clusters and moderately accurate at detecting the anxious-misery clusters. There is some debate over whether the DASS-stress measures a construct that is similar but not the same as depression and anxiety, since it nestles itself under the umbrella of the higher order negative affectivity factor (Norton, 2007). In the current study, the DASS-stress appeared to be more sensitive to the anxious-misery cluster than the fear cluster indicating some differences in the way the DASS-stress performs in regards to two clusters. One explanation is the close association between stress and worry. For example, there is evidence to suggest that individuals with non-clinical levels of frequent and uncontrollable worry also experience a high level of stress as measured by the DASS-stress (Szabo, 2011).

The high specificities and PPVs, and high sensitivities and NPVs, for the PHQ-9 and GAD-7 to detect anxious-misery indicate that screening will appropriately exclude persons without an anxious-misery disorder when adopting the recommended cut-points. Moreover, positive screens are likely to have an anxious-misery disorder requiring clinical intervention, despite lack of confirmation of a specific diagnosis. Parallel work suggests the HADS has suitable screening ROCs to detect any diagnosis of depression and anxiety from the Clinical Interview Schedule-Revised, with 85.78% sensitivity and 82.55% specificity, while the NPV was 97.63% (Palacios et al., 2016). Such work indicates that short questionnaire batteries sufficiently screen both anxiety and depression disorders in cardiovascular populations.

The results of this study should be interpreted recognizing several limitations including that the hierarchical structure of mental disorders is debated (Beesdo-Baum et al., 2009) and not always supported (Conway and Brown, 2018). This uncertainty is partly reflected in the number of cluster iterations tested here. Moreover, interpretation of the ROCs for screening purposes is closely tied to the empirical validity of such disorder structures, which have not been validated in CVD populations. A related limitation is that the current study did not include the externalizing disorder cluster (Forbes et al., 2017; Kotov et al., 2017). Since clinical trials in CVD populations typically exclude patients with externalizing disorders (e.g., alcohol/substance use disorders), the significance of these disorders, their prevalence, and prognosis are lesser known and largely examined only in epidemiological surveys (Scott et al., 2013). Moreover, the rates of externalizing disorders such as abuse and dependence were lower here than previous studies of comparable sample size (Fraguas et al., 2000). Concerning comorbidity, the MINI utilizes hierarchical exclusion rules which may lessen specific disorder comorbidity combination rates (e.g., dysthymia and major depressive disorder). The MINI has no module for specific phobias, which are less prevalent in CVD populations (Tully et al., 2014) than community estimates (Kessler et al., 2005). Given that there was a small number of OCD, PTSD, and bipolar disorder diagnoses in the current study, further investigation in diverse and larger samples of CVD patients is justified to reproduce the current findings. The timing of the assessment near an index CVD admission may spuriously inflate symptoms, particularly somatic symptoms that are commonly experienced during hospitalization and partially overlap with some mental disorders. It could also be important to test the ROCs for the PRIME-MD somatization module (Spitzer et al., 1994) against somatoform disorders. The somatoform disorders are especially prevalent in primary care populations (Piontek et al., 2018) and at least 5% of cardiac patients (Rafanelli et al., 2006). Lastly, this was a single-center design from a public hospital in Australia. Therefore, our results may not generalize to other geographic regions with diverse demographics nor to private hospitals and those serving veterans whom have a higher likelihood of PTSD (Scherrer et al., 2010).

In summary, the GAD-7 and PHQ-9 self-report scales provided sufficient ROCs to identify the anxious-misery cluster in patients recently hospitalized for CVD. The GAD-7 has additional psychometric properties making it suitable to screen for a fear cluster inclusive of OCD. Given the high likelihood of disorder comorbidity, there are potential advantages in shifting away from traditional disorder based taxonomies to an emphasis on the commonalities between disorders for screening purposes. Given that this is one of the first studies to evaluate the screening utility against internalizing disorders in patients recently hospitalized for CVD, future research should validate self-report measures against the anxious-misery and fear clusters in other larger samples.

## Members of the Champs Investigators

School of Medicine, The University of Adelaide, Adelaide, SA, Australia: John D. Horowitz, John F. Beltrame, Alison Barrett, Nathan Harrison, and Christopher Bean. Department of Cardiology, Basil Hetzel Institute, Queen Elizabeth Hospital, Woodville, SA, Australia: John F. Beltrame and Terina Selkow. Discipline of Psychiatry, Basil Hetzel Institute, Queen Elizabeth Hospital, The University of Adelaide, Adelaide, SA, Australia: Libby Markwick. University Hospital of Psychiatry and Psychotherapy, University of Münster, Münster, Germany: Bernhard T. Baune. Department of Clinical Psychology and Psychotherapy, Institute of Psychology and Education, Ulm University, Ulm Germany: Harald Baumeister. Center for Anxiety & Related Disorders, Department of Psychology, Boston University, Boston, MA, United States: Shannon Sauer-Zavala.

## Data Availability Statement

The datasets for this manuscript are not publicly available because of data management and confidentiality constraints imposed by local laws, the governing institution, and human research ethics committee. Requests to access the datasets should be directed to PT, phillip.tully@adelaide.edu.au.

## Ethics Statement

The studies involving human participants were reviewed and approved by the Human Research Ethics Committee from the Queen Elizabeth Hospital (approval #HREC/15/TQEH47). The patients/participants provided their written informed consent to participate in this study.

## Author Contributions

PT and GW: study design. PT, GW, and MG: data acquisition. MG: data analysis. MG, DT, and PT: write up. MG, DT, GW, and PT: editing the final manuscript.

## Conflict of Interest

The authors declare that the research was conducted in the absence of any commercial or financial relationships that could be construed as a potential conflict of interest.

## References

[B1] Abramowitz J. S. Foa E. B. (1998). Worries and obsessions in individuals with obsessive-compulsive disorder with and without comorbid generalized anxiety disorder. *Behav. Res. Ther.* 36 695–700. 10.1016/s0005-7967(98)00058-8 9682525

[B2] Albus C. Jordan J. Herrmann-Lingen C. (2004). Screening for psychosocial risk factors in patients with coronary heart disease-recommendations for clinical practice. *Eur. J. Cardiovasc. Prev. Rehabil.* 11 75–79. 10.1097/01.hjr.0000116823.84388.6c 15167210

[B3] Amorim P. Lecrubier Y. Weiller E. Hergueta T. Sheehan D. (1998). DSM-III-R psychotic disorders: procedural validity of the mini international neuropsychiatric interview (MINI). Concordance and causes for discordance with the CIDI. *Eur. Psychiatry* 13 26–34. 10.1016/S0924-9338(97)86748-X 19698595

[B4] Barlow D. H. Farchione T. J. Bullis J. R. Gallagher M. W. Murray-Latin H. Sauer-Zavala S. (2017). The unified protocol for transdiagnostic treatment of emotional disorders compared with diagnosis-specific protocols for anxiety disorders: a randomized clinical trial. *JAMA Psychiatry* 74 875–884. 28768327 10.1001/jamapsychiatry.2017.2164 PMC5710228

[B5] Baumeister H. Haschke A. Munzinger M. Hutter N. Tully P. J. (2015). Inpatient and outpatient costs in patients with coronary artery disease and mental disorders: a systematic review. *Biopsychosoc. Med.* 9:11. 10.1186/s13030-015-0039-z 25969694 PMC4427919

[B6] Beesdo-Baum K. Hofler M. Gloster A. T. Klotsche J. Lieb R. Beauducel A. (2009). The structure of common mental disorders: a replication study in a community sample of adolescents and young adults. *Int. J. Methods Psychiatr. Res.* 18 204–220. 10.1002/mpr.293 20024895 PMC6878418

[B7] Bohnke J. R. Lutz W. Delgadillo J. (2014). Negative affectivity as a transdiagnostic factor in patients with common mental disorders. *J. Affect. Disord.* 166 270–278. 10.1016/j.jad.2014.05.023 25012441

[B8] Brown T. A. Chorpita B. F. Korotitsch W. Barlow D. H. (1997). Psychometric properties of the depression anxiety stress scales (DASS) in clinical samples. *Behav. Res. Ther.* 35 79–89. 10.1016/s0005-7967(96)00068-x 9009048

[B9] Bunevicius A. Staniute M. Brozaitiene J. Pop V. J. Neverauskas J. Bunevicius R. (2013). Screening for anxiety disorders in patients with coronary artery disease. *Health Qual. Life Outcomes* 11:37. 10.1186/1477-7525-11-37 23497087 PMC3601013

[B10] Campbell-Sills L. Norman S. B. Craske M. G. Sullivan G. Lang A. J. Chavira D. A. (2009). Validation of a brief measure of anxiety-related severity and impairment: the overall anxiety severity and impairment scale (OASIS). *J. Affect. Disord.* 112 92–101. 10.1016/j.jad.2008.03.014 18486238 PMC2629402

[B11] Chisholm D. Sweeny K. Sheehan P. Rasmussen B. Smit F. Cuijpers P. (2016). Scaling-up treatment of depression and anxiety: a global return on investment analysis. *Lancet Psychiatry* 3 415–424. 10.1016/S2215-0366(16)30024-4 27083119

[B12] Clark L. A. Watson D. (1991). Tripartite model of anxiety and depression: psychometric evidence and taxonomic implications. *J. Abnorm. Psychol.* 100 316–336. 10.1037//0021-843x.100.3.316 1918611

[B13] Colquhoun D. M. Bunker S. J. Clarke D. M. Glozier N. Hare D. L. Hickie I. B. (2013). Screening, referral and treatment for depression in patients with coronary heart disease. *Med. J. Aust.* 198 483–484. 10.5694/mja13.10153 23682890

[B14] Conway C. C. Brown T. A. (2018). Evaluating dimensional models of psychopathology in outpatients diagnosed with emotional disorders: a cautionary tale. *Depress. Anxiety* 35 898–902. 10.1002/da.22740 29637665

[B15] Delong E. R. Delong D. M. Clarke-Pearson D. L. (1988). Comparing the areas under two or more correlated receiver operating characteristic curves: a nonparametric approach. *Biometrics* 44 837–845. 3203132

[B16] Dickens C. Cherrington A. Mcgowan L. (2012). Depression and health-related quality of life in people with coronary heart disease: a systematic review. *Eur. J. Cardiovasc. Nurs.* 11 265–275. 10.1177/1474515111430928 22457381

[B17] Eaton N. R. Krueger R. F. Markon K. E. Keyes K. M. Skodol A. E. Wall M. (2013). The structure and predictive validity of the internalizing disorders. *J. Abnorm. Psychol.* 122 86–92. 10.1037/a0029598 22905862 PMC3755742

[B18] Forbes M. K. Kotov R. Ruggero C. J. Watson D. Zimmerman M. Krueger R. F. (2017). Delineating the joint hierarchical structure of clinical and personality disorders in an outpatient psychiatric sample. *Compr. Psychiatry* 79 19–30. 10.1016/j.comppsych.2017.04.006 28495022 PMC5643220

[B19] Fraguas R. Jr. Ramadan Z. B. Pereira A. N. Wajngarten M. (2000). Depression with irritability in patients undergoing coronary artery bypass graft surgery: the cardiologist’s role. *Gen. Hosp. Psychiatry* 22 365–374. 10.1016/s0163-8343(00)00094-3 11020543

[B20] Hanley J. A. Mcneil B. J. (1982). The meaning and use of the area under a receiver operating characteristic (ROC) curve. *Radiology* 143 29–36. 10.1148/radiology.143.1.7063747 7063747

[B21] Ito M. Oe Y. Kato N. Nakajima S. Fujisato H. Miyamae M. (2015). Validity and clinical interpretability of overall anxiety severity and impairment scale (OASIS). *J. Affect. Disord.* 170 217–224. 10.1016/j.jad.2014.08.045 25259673

[B22] Kessler R. C. Chiu W. T. Demler O. Merikangas K. R. Walters E. E. (2005). Prevalence, severity, and comorbidity of 12-month DSM-IV disorders in the National Comorbidity Survey Replication. *Arch. Gen. Psychiatry* 62 617–627. 15939839 10.1001/archpsyc.62.6.617 PMC2847357

[B23] Kessler R. C. Ormel J. Petukhova M. Mclaughlin K. A. Green J. G. Russo L. J. (2011). Development of lifetime comorbidity in the World Health Organization world mental health surveys. *Arch. Gen. Psychiatry* 68 90–100. 10.1001/archgenpsychiatry.2010.180 21199968 PMC3057480

[B24] Kessler R. C. Petukhova M. Sampson N. A. Zaslavsky A. M. Wittchen H. U. (2012). Twelve-month and lifetime prevalence and lifetime morbid risk of anxiety and mood disorders in the United States. *Int. J. Methods Psychiatr. Res.* 21 169–184. 10.1002/mpr.1359 22865617 PMC4005415

[B25] Kotov R. Krueger R. F. Watson D. Achenbach T. M. Althoff R. R. Bagby R. M. (2017). The hierarchical taxonomy of psychopathology (HiTOP): a dimensional alternative to traditional nosologies. *J. Abnorm. Psychol.* 126 454–477. 10.1037/abn0000258 28333488

[B26] Kroenke K. Spitzer R. L. Williams J. B. (2001). The PHQ-9: validity of a brief depression severity measure. *J. Gen. Intern. Med.* 16 606–613. 10.1046/j.1525-1497.2001.016009606.x 11556941 PMC1495268

[B27] Krueger R. F. (1999). The structure of common mental disorders. *Arch. Gen. Psychiatry* 56 921–926. 10530634 10.1001/archpsyc.56.10.921

[B28] Ladwig K. H. Lederbogen F. Albus C. Angermann C. Borggrefe M. Fischer D. (2014). Position paper on the importance of psychosocial factors in cardiology: update 2013. *Ger. Med. Sci.* 12:Doc09. 10.3205/000194 24808816 PMC4012565

[B29] Lecrubier Y. Sheehan D. V. Weiller E. Amorim P. Bonora I. Sheehan K. H. (1997). The mini international neuropsychiatric interview (M.I.N.I.) a short diagnostic structured interview: reliability and validity according to the CIDI. *Eur. Psychiatry* 12 224–231. 10.1016/s0924-9338(97)83296-8

[B30] Lichtman J. H. Bigger J. T. Jr. Blumenthal J. A. Frasure-Smith N. Kaufmann P. G. Lespérance F. (2008). Depression and coronary heart disease: a science advisory from the American Heart Association. *Circulation* 118 1768–1775. 18824640 10.1161/CIRCULATIONAHA.108.190769

[B31] Lovibond P. F. (1998). Long-term stability of depression, anxiety, and stress syndromes. *J. Abnorm. Psychol.* 107 520–526. 10.1037//0021-843x.107.3.520 9715586

[B32] Magyar-Russell G. Thombs B. D. Cai J. X. Baveja T. Kuhl E. A. Singh P. P. (2011). The prevalence of anxiety and depression in adults with implantable cardioverter defibrillators: a systematic review. *J. Psychosom. Res.* 71 223–231. 10.1016/j.jpsychores.2011.02.014 21911099

[B33] Marker C. D. Carmin C. N. Ownby R. L. (2008). Cardiac anxiety in people with and without coronary atherosclerosis. *Depress. Anxiety* 25 824–831. 10.1002/da.20348 17597101 PMC2577372

[B34] Mineka S. Zinbarg R. (2006). A contemporary learning theory perspective on the etiology of anxiety disorders: it’s not what you thought it was. *Am. Psychol.* 61 10–26. 10.1037/0003-066x.61.1.10 16435973

[B35] Naragon-Gainey K. Prenoveau J. M. Brown T. A. Zinbarg R. E. (2016). A comparison and integration of structural models of depression and anxiety in a clinical sample: support for and validation of the tri-level model. *J. Abnorm. Psychol.* 125 853–867. 10.1037/abn0000197 27732022 PMC5117805

[B36] Norman S. B. Cissell S. H. Means-Christensen A. J. Stein M. B. (2006). Development and validation of an overall anxiety severity and impairment scale (OASIS). *Depress. Anxiety* 23 245–249. 10.1002/da.20182 16688739

[B37] Norton P. J. (2007). Depression anxiety and stress scales (DASS-21): psychometric analysis across four racial groups. *Anxiety Stress Coping* 20 253–265. 10.1080/10615800701309279 17999228

[B38] Palacios J. E. Khondoker M. Achilla E. Tylee A. Hotopf M. (2016). A single, one-off measure of depression and anxiety predicts future symptoms, higher healthcare costs, and lower quality of life in coronary heart disease patients: analysis from a multi-wave, primary care cohort study. *PLoS One* 11:e0158163. 10.1371/journal.pone.0158163 27463115 PMC4963085

[B39] Phillips A. C. Batty G. D. Gale C. R. Deary I. J. Osborn D. Macintyre K. (2009). Generalized anxiety disorder, major depressive disorder, and their comorbidity as predictors of all-cause and cardiovascular mortality: the Vietnam experience study. *Psychosom. Med.* 71 395–403. 10.1097/PSY.0b013e31819e6706 19321850

[B40] Piontek K. Shedden-Mora M. C. Gladigau M. Kuby A. Löwe B. (2018). Diagnosis of somatoform disorders in primary care: diagnostic agreement, predictors, and comparisons with depression and anxiety. *BMC Psychiatry* 18:361. 10.1186/s12888-018-1940-3 30419878 PMC6233530

[B41] Plummer F. Manea L. Trepel D. Mcmillan D. (2016). Screening for anxiety disorders with the GAD-7 and GAD-2: a systematic review and diagnostic metaanalysis. *Gen. Hosp. Psychiatry* 39 24–31. 10.1016/j.genhosppsych.2015.11.005 26719105

[B42] Rafanelli C. Roncuzzi R. Milaneschi Y. (2006). Minor depression as a cardiac risk factor after coronary artery bypass surgery. *Psychosomatics* 47 289–295. 10.1176/appi.psy.47.4.289 16844886

[B43] Robertson N. Javed N. Samani N. J. Khunti K. (2008). Psychological morbidity and illness appraisals of patients with cardiac and non-cardiac chest pain attending a rapid access chest pain clinic: a longitudinal cohort study. *Heart* 94:e12. 10.1136/hrt.2006.100537 17540685

[B44] Roest A. M. De Jonge P. Lim C. W. W. Stein D. J. Al-Hamzawi A. Alonso J. (2017). Fear and distress disorders as predictors of heart disease: a temporal perspective. *J. Psychosom. Res.* 96 67–75. 10.1016/j.jpsychores.2017.03.015 28545795 PMC5674522

[B45] Roest A. M. Martens E. J. Denollet J. De Jonge P. (2010). Prognostic association of anxiety post myocardial infarction with mortality and new cardiac events: a meta-analysis. *Psychosom. Med.* 72 563–569. 10.1097/PSY.0b013e3181dbff97 20410247

[B46] Rothman K. J. (1990). No adjustments are needed for multiple comparisons. *Epidemiology* 1 43–46. 10.1097/00001648-199001000-00010 2081237

[B47] Rutledge T. Reis V. A. Linke S. E. Greenberg B. H. Mills P. J. (2006). Depression in heart failure a meta-analytic review of prevalence, intervention effects, and associations with clinical outcomes. *J. Am. Coll. Cardiol.* 48 1527–1537. 17045884 10.1016/j.jacc.2006.06.055

[B48] Scherrer J. F. Chrusciel T. Zeringue A. Garfield L. D. Hauptman P. J. Lustman P. J. (2010). Anxiety disorders increase risk for incident myocardial infarction in depressed and nondepressed Veterans Administration patients. *Am. Heart J.* 159 772–779. 10.1016/j.ahj.2010.02.033 20435185

[B49] Scott K. M. De Jonge P. Alonso J. Viana M. C. Liu Z. O’neill S. (2013). Associations between DSM-IV mental disorders and subsequent heart disease onset: beyond depression. *Int. J. Cardiol.* 168 5293–5299. 10.1016/j.ijcard.2013.08.012 23993321 PMC3886238

[B50] Scott K. M. Von Korff M. Alonso J. Angermeyer M. C. Bromet E. Fayyad J. (2009). Mental-physical co-morbidity and its relationship with disability: results from the World Mental Health Surveys. *Psychol. Med.* 39 33–43. 10.1017/S0033291708003188 18366819 PMC2637813

[B51] Serber E. R. Todaro J. F. Tilkemeier P. L. Niaura R. (2009). Prevalence and characteristics of multiple psychiatric disorders in cardiac rehabilitation patients. *J. Cardiopulm. Rehabil. Prev.* 29 161–168. 10.1097/HCR.0b013e3181a33365 19471134 PMC4445736

[B52] Sheehan D. V. Lecrubier Y. Harnett-Sheehan K. Janavs J. Weiller E. Bonora L. I. (1997). Reliability and validity of the mini international neuropsychiatric interview (M.I.N.I.): according to the SCID-P. *Eur. Psychiatry* 12 232–241. 10.1016/s0924-9338(97)83297-x

[B53] Sheehan D. V. Lecrubier Y. Sheehan K. Amorim P. Janavs J. Weiller E. (1998). The mini-international neuropsychiatric interview (M.I.N.I.): the development and validation of a structured diagnostic psychiatric interview for DSM-IV and ICD-10. *J. Clin. Psychiatry* 59 22–33. 9881538

[B54] Slade T. Johnston A. Oakley Browne M. A. Andrews G. Whiteford H. (2009). National survey of mental health and wellbeing: methods and key findings. *Aust. N. Z. J. Psychiatry* 43 594–605. 10.1080/00048670902970882 19530016

[B55] Slade T. Watson D. (2006). The structure of common DSM-IV and ICD-10 mental disorders in the Australian general population. *Psychol. Med.* 36 1593–1600. 10.1017/s0033291706008452 16882356

[B56] Sommaruga M. Angelino E. Della Porta P. Abatello M. Baiardo G. Balestroni G. (2018). Best practice in psychological activities in cardiovascular prevention and rehabilitation: position paper. *Monaldi Arch. Chest Dis.* 88:966. 10.4081/monaldi.2018.966 29962189

[B57] Spitzer R. L. Kroenke K. Williams J. B. Lowe B. (2006). A brief measure for assessing generalized anxiety disorder: the GAD-7. *Arch. Intern. Med.* 166 1092–1097. 16717171 10.1001/archinte.166.10.1092

[B58] Spitzer R. L. Williams J. B. Kroenke K. Linzer M. Degruy F. V. III Hahn S. R. (1994). Utility of a new procedure for diagnosing mental disorders in primary care. The PRIME-MD 1000 study. *JAMA* 272 1749–1756. 10.1001/jama.272.22.1749 7966923

[B59] Szabo M. (2011). The emotional experience associated with worrying: anxiety, depression, or stress? *Anxiety Stress Coping* 24 91–105. 10.1080/10615801003653430 20198520

[B60] Thombs B. D. De Jonge P. Coyne J. C. Whooley M. A. Frasure-Smith N. Mitchell A. J. (2008). Depression screening and patient outcomes in cardiovascular care: a systematic review. *JAMA* 300 2161–2171. 10.1001/jama.2008.667 19001627

[B61] Thombs B. D. Roseman M. Coyne J. C. De Jonge P. Delisle V. C. Arthurs E. (2013). Does evidence support the american heart association’s recommendation to screen patients for depression in cardiovascular care? An updated systematic review. *PLoS One* 8:e52654. 10.1371/journal.pone.0052654 23308116 PMC3538724

[B62] Tully P. J. Cosh S. M. Baumeister H. (2014). The anxious heart in whose mind? A systematic review and meta-regression of factors associated with anxiety disorder diagnosis, treatment and morbidity risk in coronary heart disease. *J. Psychosom. Res.* 77 439–448. 10.1016/j.jpsychores.2014.10.001 25455809

[B63] Tully P. J. Penninx B. W. (2012). Depression and anxiety among coronary heart disease patients: can affect dimensions and theory inform diagnostic disorder based screening? *J. Clin. Psychol.* 68 448–461. 10.1002/jclp.21828 22308051

[B64] Tully P. J. Turnbull D. Horowitz J. D. Beltrame J. F. Selkow T. Baune B. T. (2016). Cardiovascular health in anxiety or mood problems study (CHAMPS): study protocol for a randomized controlled trial. *Trials* 17:18. 10.1186/s13063-015-1109-z 26754447 PMC4707770

[B65] Tully P. J. Wardenaar K. J. Penninx B. W. (2015a). Operating characteristics of depression and anxiety disorder phenotype dimensions and trait neuroticism: a theoretical examination of the fear and distress disorders from the Netherlands study of depression and anxiety. *J. Affect. Disord.* 174 611–618. 10.1016/j.jad.2014.12.045 25577156

[B66] Tully P. J. Winefield H. R. Baker R. A. Denollet J. Pedersen S. S. Wittert G. A. (2015b). Depression, anxiety and major adverse cardiovascular and cerebrovascular events in patients following coronary artery bypass graft surgery: a five year longitudinal cohort study. *Biopsychosoc. Med.* 9:14. 10.1186/s13030-015-0041-5 26019721 PMC4445298

[B67] Vos T. Flaxman A. D. Naghavi M. Lozano R. Michaud C. Ezzati M. (2012). Years lived with disability (YLDs) for 1160 sequelae of 289 diseases and injuries 1990-2010: Global Burden of Disease Study 2010. *Lancet* 380 2163–2196. 10.1016/S0140-6736(12)61729-2 23245607 PMC6350784

[B68] Waszczuk M. A. Kotov R. Ruggero C. Gamez W. Watson D. (2017). Hierarchical structure of emotional disorders: from individual symptoms to the spectrum. *J. Abnorm. Psychol.* 126 613–634. 10.1037/abn0000264 28471212

[B69] Watkins L. L. Koch G. G. Sherwood A. Blumenthal J. A. Davidson J. R. O’connor C. (2013). Association of anxiety and depression with all-cause mortality in individuals with coronary heart disease. *J. Am. Heart Assoc.* 2:e000068. 10.1161/JAHA.112.000068 23537805 PMC3647264

[B70] Watson D. (2005). Rethinking the mood and anxiety disorders: a quantitative hierarchical model for DSM-V. *J. Abnorm. Psychol.* 114 522–536. 10.1037/0021-843x.114.4.522 16351375

[B71] Watson D. (2009). Differentiating the mood and anxiety disorders: a quadripartite model. *Annu. Rev. Clin. Psychol.* 5 221–247. 10.1146/annurev.clinpsy.032408.153510 19327030

